# Responses of the Endophytic Bacterial Communities of *Juncus acutus* to Pollution With Metals, Emerging Organic Pollutants and to Bioaugmentation With Indigenous Strains

**DOI:** 10.3389/fpls.2018.01526

**Published:** 2018-10-18

**Authors:** Evdokia Syranidou, Sofie Thijs, Marina Avramidou, Nele Weyens, Danae Venieri, Isabel Pintelon, Jaco Vangronsveld, Nicolas Kalogerakis

**Affiliations:** ^1^School of Environmental Engineering, Technical University of Crete, Chania, Greece; ^2^Centre for Environmental Sciences, Hasselt University, Diepenbeek, Belgium; ^3^Laboratory of Cell Biology and Histology, University of Antwerp, Antwerp, Belgium; ^4^Department of Chemical Engineering, American University of Sharjah, Sharjah, United Arab Emirates

**Keywords:** wetland plant, *J. acutus*, endophytic bacterial community, metals, emerging organic contaminants

## Abstract

Plants and their associated bacteria play a crucial role in constructed wetlands. In this study, the impact of different levels of pollution and bioaugmentation with indigenous strains individually or in consortia was investigated on the composition of the endophytic microbial communities of *Juncus acutus*. Five treatments were examined and compared in where the wetland plant was exposed to increasing levels of metal pollution (Zn, Ni, Cd) and emerging pollutants (BPA, SMX, CIP), enriched with different combinations of single or mixed endophytic strains. High levels of mixed pollution had a negative effect on alpha diversity indices of the root communities; moreover, the diversity indices were negatively correlated with the increasing metal concentrations. It was demonstrated that the root communities were separated depending on the level of mixed pollution, while the family *Sphingomonadaceae* exhibited the higher relative abundance within the root endophytic communities from high and low polluted treatments. This study highlights the effects of pollution and inoculation on phytoremediation efficiency based on a better understanding of the plant microbiome community composition.

## Introduction

Constructed wetlands (CWs) are a promising alternative for treating various chemical compounds and for preventing their dispersion in the environment (Verlicchi and Zambello, 2014). They are engineered, state of the art sustainable systems used to treat effluents rich in pharmaceutical and personal care products by exploiting plant-bacteria interactions in combination with physicochemical processes (Zhang et al., 2014). Recently, many studies have focused on investigating the efficiency of wetland plants to remove EOCs in hydroponic systems and the potential effects of these compounds on the plant physiological status (Dordio et al., 2011; Liu et al., 2013; Christofilopoulos et al., 2016). They are also efficient in removing metals from various influents such as industrial wastewater (Khan et al., 2009), landfill leachate (A et al., 2017), and acid mine drainage (Wu et al., 2015).

In such systems, the selection of both, the appropriate plant species and their associated rhizospheric and endophytic microbiota, significantly influence the performance of the CW (Guittonny-Philippe et al., 2014; Li et al., 2014). Rhizospheric microorganisms can enhance xenobiotic transformations *ex planta* and improve nutrient uptake, while the endophytic community is important for transforming the organic compounds *in planta*, thus reducing their toxicity and evapotranspiration of water-soluble and volatile compounds (Barac et al., 2004; Taghavi et al., 2005; Afzal et al., 2014). The contribution of plant-associated microorganisms to metal phytoremediation has also been highlighted through promoting plant growth in metal polluted areas, influencing metal uptake and translocation, and increasing the metal bioavailability by secretion of ligands and organic acids (Sessitsch et al., 2013; Ma et al., 2016a).

Only a few studies attempted to explore the bacterial communities associated with wetland plants and even fewer to describe the responses of such communities to mixed and highly polluted environments (Su et al., 2015; Zhao et al., 2015; Zhang D. et al., 2016; Syranidou et al., 2017b). Moreover, information relevant to the impacts of pollutants on the endophytic bacteria of wetland plants is scarce. In such complicated environments, it is unclear whether the type and level of pollution, the plant species, the application of biostimulant bacteria, or a multifactor combination influence the phytoremediation potential and underlying endophytic assemblages. However, in order to improve the performance of CWs, it is important to address these questions.

Previously, we showed that inoculation of the wetland plant *J. acutus* with a selected endophytic bacterial consortium removed emergent contaminants and metals faster and more efficiently compared to non-inoculated plants (Syranidou et al., 2016). Moreover, bioaugmentation with a tailored endophytic consortium enhanced phytoremediation efficacy, and the microbes seemed to alleviate the stress induced by the pollutants, especially at the high concentration treatments. The consortium consisted of strains tolerant to metals (Zn, Ni, Cd) and emerging contaminants (BPA, CIP, SMX) and they have been characterized as potential degraders of these contaminants (Syranidou et al., 2017b). Because insights in this important part was lacking completely, we decided to perform a deeper analyses and study the total endophytic community response. In this study, the effect of mixed pollutants and bioaugmentation with single indigenous endophytic strains or in a consortium was addressed on the endophytic bacterial community of *J. acutus*, under different levels of mixed pollutants.

## Materials and Methods

### Experimental Design

Three endophytic strains (leaf B1- *Sphingomonas* sp. U33, root B2- *Bacillus* sp. R12, root B3- *Ochrobactrum* sp. R24) (Syranidou et al., 2016) were inoculated separately and as a consortium to beakers (*n* = 10 for every treatment) with *J. acutus* plants. One week later, two different concentrations of metals (Zn, Ni, Cd), bisphenol-A (BPA) and two antibiotics [ciprofloxacin (CIP) and sulfamethoxazole (SMX)] were added. More specifically, 50 μg L^-1^ CIP, 250 μg L^-1^ SMX, 5 mg L^-1^ BPA, 200 mg L^-1^ Zn, 20 mg L^-1^ Ni, and 1 mg L^-1^ Cd were added to the low concentration treatments and 100 μg L^-1^ CIP, 500 μg L^-1^ SMX, 10 mg L^-1^ BPA, 400 mg L^-1^ Zn, 40 mg L^-1^ Ni, and 2 mg L^-1^ Cd were used in the high concentration treatments. Further, in four treatments no pollutants were added in order to investigate the potential effects of inoculation on the endophytic community in the absence of pollutants. A two-factor design study was followed with factor 1 pollutant concentration (three levels, zero, low, high), and factor 2 bioaugmentation treatments (five levels, no inoculation, strain 1, strain 2, strain 3, consortium). In total, there were five different treatments (one non-inoculated control and four bioaugmented treatments) concerning the inoculation effect and three different concentrations of the mixture of pollutants (one without pollutants-NO, one low concentration-LC and one high concentration-HC) concerning the pollution effect. The experiment lasted for 21 days and was irrigated with 50 mL tap water every week. A schematic representation of the experimental design in provided in **Supplementary Figure S1**.

### Sample Collection

Fresh root (0.3 g) and leaf (1 g) tissue samples (*n* = 3 for every treatment) were collected for DNA extraction at the end of the experiment. In order to sterilize the outer surface, the plant plants were immersed in 70% ethanol for 30 s and subsequently in 2% NaClO solution supplemented with one droplet Tween 80 per 100 mL solution for 10 min. Subsequently, they were rinsed three times with sterile distilled water for 1 min and 100 μL of the last rinsing solution were streaked on 869 plates (Mergeay et al., 1985) and incubated for 7 days at 30°C to verify the surface sterility. The maceration of the plant samples was performed with liquid nitrogen and total DNA was extracted using the Invisorb^®^ Spin Plant Mini Kit (STRATEC Molecular GmbH, Berlin, Germany).

### 16S rRNA Gene Amplicon Libraries Preparation

The forward 799F primer (AACMGGATTAGATACCCKG) and the reverse primer 1193R (ACGTCATCCCCACCTTCC) were used for the amplification of the V5–V7 hypervariable region of the bacterial 16S rRNA gene, producing a ∼400 bp fragment (Schlaeppi et al., 2014). The primer pair was selected based on the 2 bp mismatch at the 3′-end of the 799F primer with the chloroplastidal DNA. After the first PCR, the bacterial DNA was selected over the not always present mitochondrial DNA (approximately 800 bp). The bacterial amplicons were collected and purified from agarose with the QIAquick gel extraction kit (Qiagen, Venlo, Netherlands). For multiplexed pyrosequencing, a sample-specific 10 bp barcode (MID) was fused to the forward primer, followed by the key and a Lib-L Adaptor A sequence. Every PCR reaction contained 1 × FastStart High Fidelity Reaction buffer (Roche) with 1.8 mM MgCl_2_ (Roche), 200 μM of each dNTP (Roche), 250 nM forward primer, 250 nM reverse primer, 1.25 U FastStart High Fidelity Taq DNA polymerase (Roche), 1 μL DNA template and RNase free water until a total volume of 25 μL. The PCR conditions were: an initial denaturation step of 2.5 min at 95°C, 35 (1st PCR) or 20 (2nd PCR) cycles of denaturation of 1 min at 94°C, annealing for 40 s at 53°C and extension for 40 s at 72°C, and a final extension step of 7 min at 72°C. The bacterial amplicons produced by the second PCR were purified and the concentration of purified DNA was determined with the Quant-iT Picogreen dsDNA assay kit (Life Technologies Europe, Ghent, Belgium) according to the manufacture’s protocol. Equimolar mixtures of different samples were prepared. For checking the amplicons, 1 μL of the library was loaded on a DNA-chip (DNA 1000 kit, Agilent Technologies, Diegem, Belgium) and analyzed on a 2100 Bioanalyzer (Agilent Technologies, Diegem, Belgium). The libraries were clonally amplified using the emPCR Lib-L kit and then sequenced using the Roche 454 GS-FLX Plus Life Sciences Genome Sequencer at Macrogen, Seoul, South Korea.

### 16s rRNA Gene Sequences Analysis

Bacterial amplicons were quality filtered and trimmed using the DADA2 v1.8 pipeline in R v3.4 (Callahan et al., 2016) with the adapted following read settings: filtering criteria (max *N* = 0, Max EE = 2, TruncQ = 2, trimming of the first 15 bp, and fixed trunclength of 300 bp). Prediction of Absolute sequence variants (ASVs) was performed with DADA2, homopolymer gap penalty was set to -1 and band size was equal to 32, followed by de novo chimera removal. The high quality reads were classified against the SILVA v132 training dataset (Quast et al., 2013; Yilmaz et al., 2014; Glöckner et al., 2017) using the naive Bayesian classifier method in DADA2. The standard flowgram format (SFF) files were deposited in the NCBI Sequence Read Archive (SRA) under the accession number SRP158657.

### Metal Analysis in Plant Parts

*Juncus acutus* roots and leaves (*n* = 10 for every treatment) were washed with tap water (3×) followed by washing with distilled water in order to remove any adhered particles. Next, they were dried at 50°C for 4 days. Dried plant samples (0.2 g) were digested with 9 mL HNO_3_ (>69%, Sigma-Aldrich) and diluted with ultrapure water and centrifuged. Supernatants were subsequently filtered (0.45 μm, Whatman), diluted at 1:10 (v/v) with ultrapure water and analyzed by ICP-MS (ICP-MS 7500cx coupled with Autosampler Series 3000, both from Agilent Technologies).

### Colonization and Distribution of Endophytes Within Host Plants

Strains were tagged with fluorescent proteins as previously described (Sánchez-López et al., 2018) in order to monitor their colonization to the host plant under similar experimental conditions. The labeled strains were inoculated (10^9^ cells mL^-1^) to plants with and without addition of pollutants. Their colonization efficiency was investigated with a confocal laser microscope Ultra VIEW VoX, PerkinElmer (Zaventem, Belgium) using an excitation wavelength of 561 nm (red) for mCherry, and 405 (Dapi) for plant cell walls while the confocal pictures were analyzed using ImageJ software and Amira 3D visualization software version 6.1.0 (FEI Visualization Sciences Group, Hillsboro, OR, United States) as previously described (Sánchez-López et al., 2018).

### PCR and qPCR

The occurrence of various ARGs such as the sulfamethoxazole (*sul*I) and ciprofloxacin resistance genes [*qnrA, qnrS* and *aac(6′)-Ib*] was screened via polymerase chain reaction (PCR) detection assays. The primers sul1-F (5′-CTT CGA TGA GAG CCG GCG GC-3′) and sul1-R (5′-GCA AGG CGG AAA CCC GCG CC-3′) (Jacobs and Chenia, 2007), aac-F (5′-TTGCGA TGCTCTATGAGTGGCTA-3′) and aac-R (5′-CTCGAATGCCTGGC GTGTTT-3′) (Park et al., 2006), qnrA-F (5′-GAT AAA GTT TTT CAG CAA GAG G-3′) and qnrA-R (5′-ATC CAG ATC GGC AAA GGT TA-3′) and qnrS-F (5′-GTA TAG AGT TCC GTG CGT GTG A-3′) and qnrS-R (5′-GGT TCG TTC CTA TCC AGC GAT T-3′) (Mao et al., 2015) were used for the detection of *sul*I, *aac(6*′*)-lb-cr*, *qnrA*, and *qnrS* genes, respectively. PCR assays were performed as previously described (Park et al., 2006; Jacobs and Chenia, 2007; Mao et al., 2015). Since only the *sul*I was detected in the endophytic communities, the abundance of this gene was estimated using a StepOne Plus System (Applied Biosystems Inc., Foster City, CA, United States). qPCR reaction was performed in a 20 μL volume mixture and conducted in 96 well plates containing 10 μL of SYBR Green Dye (Applied Biosystems), 0.2 μM of each primer and 2 μL of template DNA. The detailed protocol was as follows: 94°C for 5 min, followed by 35 cycles of 94°C for 30 s, annealing at 55°C for 60 s and 72°C for 2 min. All samples and standards were amplified in triplicates. For the standard curve, six-fold serial dilution of the *sul*I gene isolated from the B3 endophytic strain was performed. The amplification efficiency and coefficient (*r*^2^) was 110% and 0.98, respectively. Melting-curve and a 1.5% agarose gel were used for assuring the specificity of the products.

### Statistical Analysis

Statistical analysis was performed with R v3.3.2 (R Development Core Team, 2016). Differences in the metal concentrations and alpha diversity indices in different plant parts among the treatments were estimated with an analysis of variance (two-way ANOVA). After detecting significant differences, a multiple Tukey comparison test was performed. Correlation analysis between the diversity indices and the different metal concentrations was performed in R [package: Hmisc and corrplot (Wei and Simko, 2017)], based on Pearson’s product moment correlation coefficient. Processed amplicon sequencing data were analyzed using the Bioconductor package phyloseq v1.19.1 (McMurdie and Holmes, 2013). The raw data was used prior to alpha-diversity analyses. For beta-diversity analyses, an inclusion threshold of 2% prevalence was used, data was normalized by total sum scaling and expressed in relative abundance %, followed by a *log*(1+x) transformation. Community dissimilarities were represented by non-metric multidimensional scaling (NMDS) using the Bray–Curtis distance and by Principal coordinate analysis (PCoA) using weighted and unweighted UniFrac distance.

To evaluate the similarity of community assemblages among the samples, PERMANOVA (R-vegan function Adonis) was performed onto the Bray–Curtis dissimilarity matrix with *n* = 999 permutations. Mantel test analysis was performed in order to detect any significant correlation between the presence of metals under different levels of mixed pollution and endophytic bacteria composition using the Bray–Curtis dissimilarity matrix. LEfSe [Linear Discriminant Analysis (LDA) Effect Size] was used to detect the bacterial taxonomic biomarkers across the different treatments (Segata et al., 2011). In addition, differentially abundant taxa were identified using DESeq2 v1.14.1 analyses in R (Love et al., 2014). The Venn diagrams were generated according to Wang et al. (2016).

## Results

The effects of bioaugmentation with endophytic bacteria on the efficiency of phytoremediation of mixed pollutants was investigated with promising results (Syranidou et al., 2016). In this study, the effects of mixed pollutants and bioaugmentation on the root and leaf endophytic communities were investigated in depth to obtain a better understanding of the interactions between microbes and their host plants during wastewater treatment.

### Metal Uptake by Plants

The metal concentrations in *J. acutus* roots and leaves tended to increase in the treatments with high concentrations of mixed pollution (**Supplementary Figures S2–S4**) while roots accumulated higher concentrations of metals compared to the leaves in every treatment. Bioaugmentation through inoculation with indigenous endophytic bacteria increased Zn concentrations in roots and leaves and two-way ANOVA revealed significant effects of the level of pollution as well as of inoculation on the metal concentrations in plants. When exposed to 200 mg L^-1^ Zn, all the inoculants significantly enhanced the phytoextraction capacity of the plants compared to the non-inoculated ones. At elevated Zn concentrations, plants inoculated with strain B1, B3 and the consortium accumulated significantly higher amounts of Zn in the roots while B1, B2, and B3 inoculated plants accumulated significantly more Zn in the leaves in comparison to the non-inoculated plants, indicating an increase in the translocation factor. With respect to nickel, the beneficial effects of bioaugmentation on the phytoextraction capacity of *J. acutus* were less pronounced. Nevertheless, significant effects on plant Ni concentration were observed depending on the metal concentrations they were exposed to. At low Ni concentrations, the B1 inoculated plants accumulated significantly more Ni in the roots in comparison to the non-inoculated plants while a significantly higher Ni concentration was detected in the leaves of the plants inoculated with the consortium. When exposed to 40 mg L^-1^ Ni, a significant increase in the Ni concentration in the roots of B1 inoculated plant was observed in comparison to the non-inoculated plants. The B1, B2 and B3 inoculated plants contained significantly higher Ni concentrations in their leaves in comparison to the non-inoculated plants.

Cadmium was not detected in leaves of all plants regardless of the initial exposure concentration and inoculation effort. In roots, there was no significant difference in Cd accumulation capacity of inoculated and non-inoculated plants at low and high Cd exposure.

### Responses of Endophytic Bacterial Communities

In order to investigate the interactions between the host plant and its associated endophytic microbial community during phytoremediation of mixed polluted water as well as in case of bioaugmentation with indigenous endophytic strains, the total DNA was extracted from *J. acutus* leaves and roots and was analyzed using high-throughput sequencing. A total of 358035 16S rRNA gene sequences were obtained from 66 samples, with average read length of 353 bp. After the sequence quality filtering, denoising and removing of all chimeric sequences 63 samples remained with 231202 assembled high-quality sequences and 2680 ASVs recorded.

**FIGURE 1:**
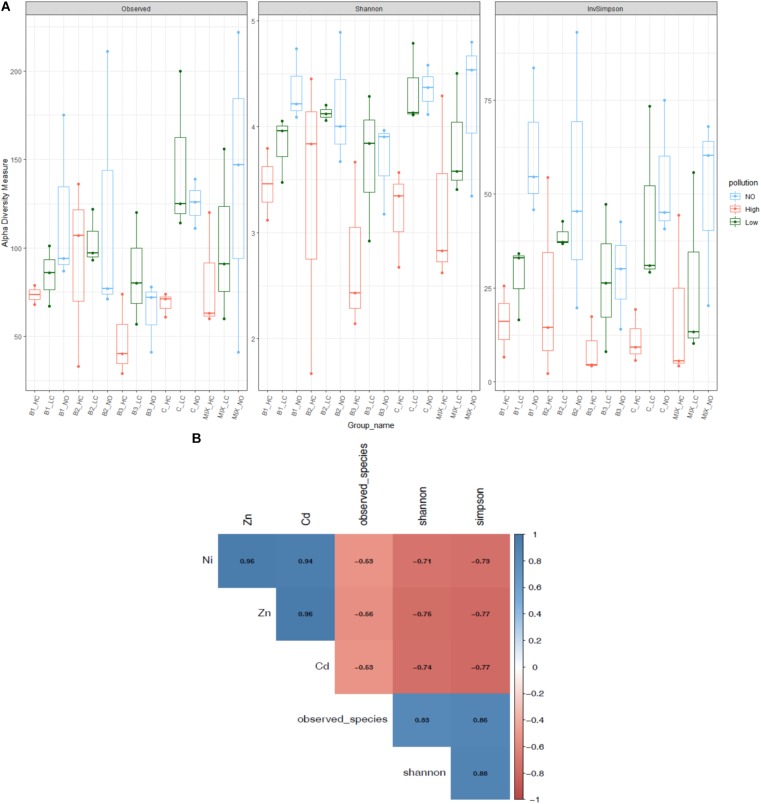
Alpha diversity indices of root endophytic communities among the different bioaugmented treatments **(A)** and the correlation of them with the metal concentration in the roots (the significant correlation coefficients are indicated) **(B)**. The scale colors denote whether the correlation is positive (closer to 1, blue circles) or negative (closer to –1, red circles) between the diversity indices and metal concentrations.

High levels of mixed pollution had a negative effect on alpha diversity indices of the root communities (**Figure 1A**). In particular, pollution significantly lowered the Shannon diversity (*F*: 8.8, *p* = 0.001) as well as all the indices. Significant differences were detected between the indices of the communities from highly polluted with the communities of plants exposed to low pollution and no pollution, while no significant differences were noticed between the low and non-polluted treatments. The Simpson diversity index significantly differed in communities exposed to low pollution and no pollution. The effect of various inoculants on Shannon diversity was minimal (*F*: 1.1, *p* = 0.37), as well as on all the indices.

Correlation analysis revealed that the number of observed species, phylogenetic diversity, and Shannon and Simpson diversity of the root communities negatively correlated with increased concentrations of metals in roots (**Figure 1B**). The number of observed species was negatively affected by nickel, zinc and cadmium concentrations in the root compartment (Ni: *p* < 10^-4^, *r* = -0.53, Zn: *p* < 10^-4^, *r* = -0.56, Cd: *p* < 10^-4^, *r* = -0.53), as well as the Simpson diversity index (Ni: *p* < 10^-4^, *r* = -0.73, Zn: *p* < 10^-4^, *r* = -0.77, Cd: *p* < 10^-4^, *r* = -0.77). Similarly, the increased metal concentrations decreased significantly the Shannon diversity (Ni: *p* < 10^-4^, *r* = -0.71, Zn: *p* < 10^-4^, *r* = -0.76, Cd: *p* < 10^-4^, *r* = -0.74).

**FIGURE 2:**
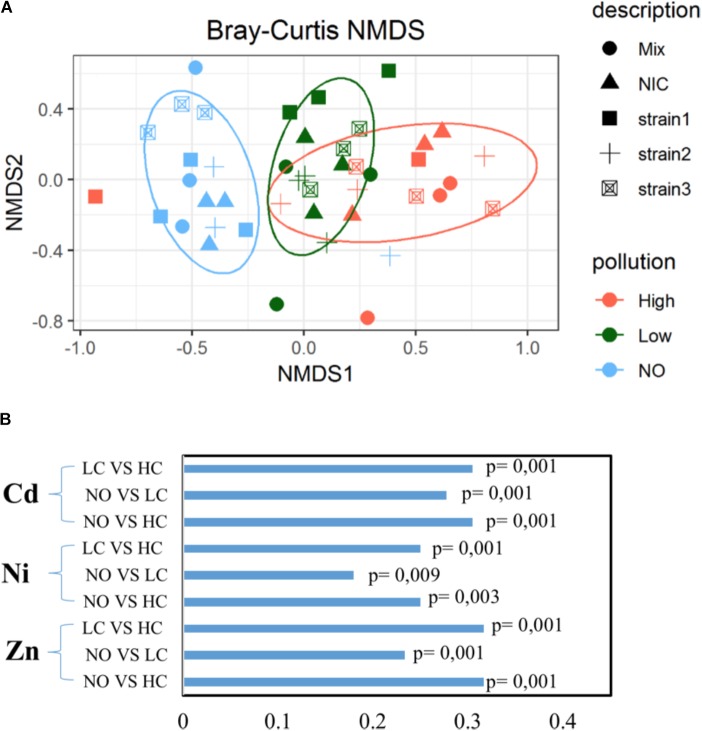
Similarity of the root endophytic communities across a mixed pollution gradient **(A)**. Distance between the samples is based on similarity of the community composition visualized in non-metric multidimensional scaling (NMDS). **(B)** Phylogenetic correlation of root bacterial communities across metal gradient in roots using the Mantel test (999 permutations).

The *J. acutus* root endophytic communities seemed to alter in function of the level of pollution. As seen in **Figure 2A**, separate groups are formed, while this separation is statistically significant (*p* = 0.001). Each group involved samples of plants exposed to the same concentration of the mixture of metals and emerging organic pollutants (High, Low, NO), indicating an induced shift in the root community composition. Root endophytic communities from the non-polluted treatments were clearly different from the others, whereas a slight overlap was noticed between communities of plants exposed to low and high pollution. With respect to the composition of the leaf endophytic community, no clear pattern was detected (**Supplementary Figure S5**). It appears that almost all samples are grouped together regardless of the level of pollution or inoculation effort.

Mantel correlation coefficients indicated that significant relationships existed between root communities of plants exposed to different concentrations of metals (**Figure 2B**). When the root communities from the different inoculation treatments were investigated separately, different correlations between endophytic assemblages and the exposure concentration of pollutants were revealed. The root endosphere of B2 inoculated plants was significantly correlated with the increasing metal concentrations in this plant compartment, while no association was observed between the other endophytic communities and each metal, indicating treatment-specific responses.

### Endophytic Community Composition

**FIGURE 3:**
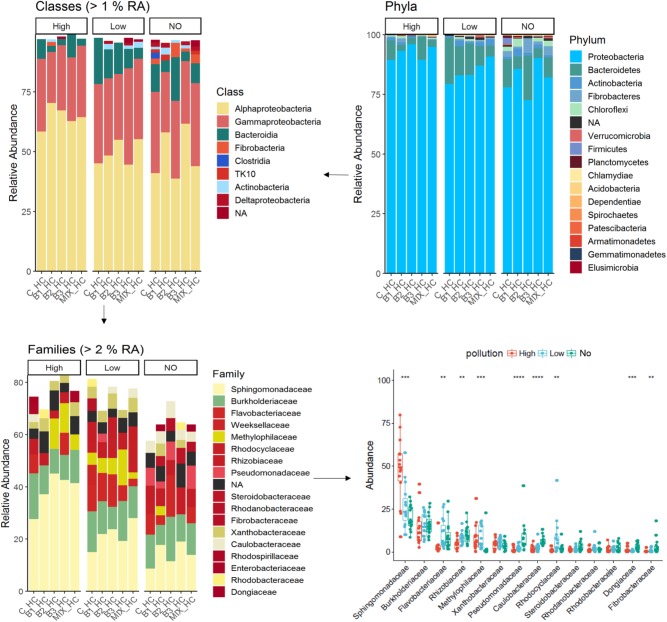
Overview of the relative populations of root endophytes with respect to phylum, class, and family under the different level of pollution.

In this study, 16 phyla were detected in the endosphere of *J. acutus*; overall the phylum Proteobacteria dominated the endophytic bacterial community. The root community consisted mainly of Alphaproteobacteria followed by Gamma-proteobacteria, and members of Bacteroidia, Fibrobacteria, and Actinobacteria (**Figure 3**).

The abundance of Proteobacteria was significantly increased in root communities exposed to elevated concentrations of mixed pollution, while the phyla Bacteroidetes and Fibrobacteres significantly decreased. In accordance, the abundance of Alphaproteobacteria was significantly increased in roots of plants grown in high polluted treatments in comparison to unpolluted ones, while the abundance of Fibrobacteria, Bacteroidia, and Deltaproteobacteria was significantly decreased. Within the root endophytes, those affiliated with the order Sphingomonadales were enriched in the *J. acutus* roots exposed to high pollution whereas those affiliated with Flavobacteriales were enriched in roots exposed to low pollution. The family *Sphingomonadaceae* exhibits the higher relative abundance within the root endophytic communities from high and low polluted treatments while the abundance of the families *Pseudomonadaceae* and *Rhizobiaceae* is higher in roots of plants grown in absence of pollutants.

**FIGURE 4:**
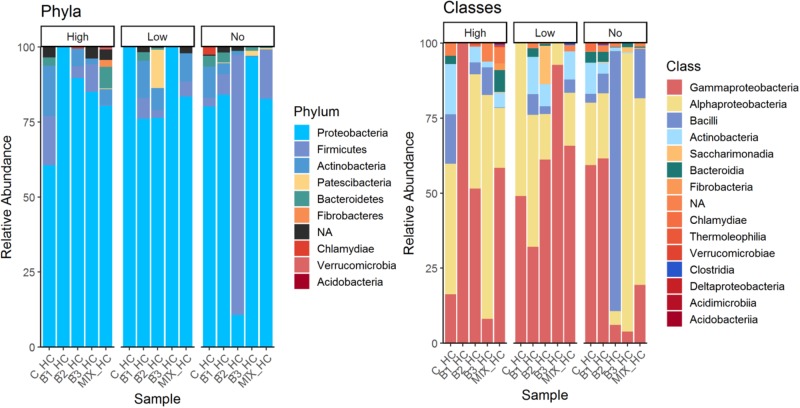
Overview of the relative populations of leaf endophytes with respect to phylum and class under the different level of pollution.

**FIGURE 5:**
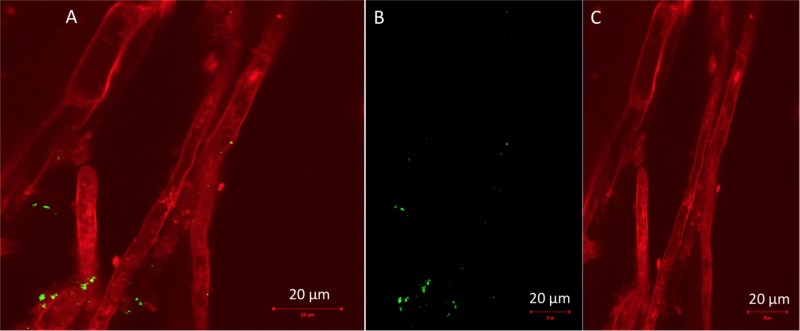
Confocal laser scanning micrographs of **(A)** gfp-tagged B3 strain colonizing roots of *Juncus acutus*, **(B)** solution with live gfp-labeled B3 bacteria, and **(C)** root tissue autofluorescence.

The phylum Proteobacteria dominated almost all leaf communities; in general endophytic leaf community abundance profiles showed more variable patterns compared to the root communities. The most abundant classes were Alphaproteobacteria followed by Gamma-proteobacteria, and members of Bacteroidia, Fibrobacteria and Actinobacteria, while Bacilli and Saccharimonadia exhibited high relative abundance (**Figure 4**). It is important to notice that the class Bacilli dominated the leaf community of B2 inoculated plants that were not exposed to pollution which is in accordance with the inoculant. Similarly, the relative abundance of the B1 strain was higher in the leaf endophytic communities of unexposed plants in comparison to exposed ones. As seen in **Figure 5**, the endophytic stain B3 could efficiently colonize the roots surface of *J. acutus* in the presence of mixed pollution.

**FIGURE 6:**
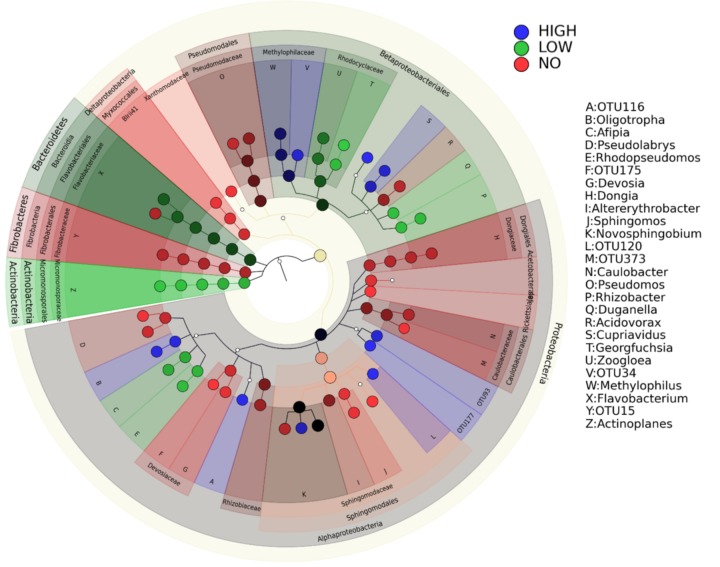
Biomarkers of the endophytic community of roots exposed to high and low concentration of pollutants and of the roots in unpolluted treatments. LEfSe was used to validate the statistical significance and the size effect of the differential abundances of taxa (Kruskal–Wallis and Wilcoxon rank-sum *p* < 0.05 and LDA score >3.5). In the cladogram, the class, order, and family are presented and the genus is represented using letters.

**FIGURE 7:**
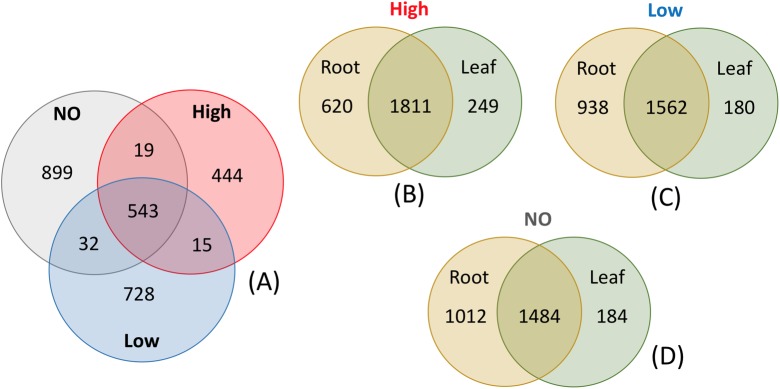
Venn diagrams of root endophytic communities exposed to a mixed pollution gradient **(A)**, and endophytic communities of *J. acutus* exposed to high **(B)**, low **(C)** pollution as well as no pollution **(D)**.

Changes in the community members were observed in response to increasing levels of pollution (**Figure 6**). The root communities from plants exposed to different concentrations of mixed pollution were compared in order to identify the potential indicator taxa. Only 85 root ASVs (LEfSE, *p* < 0.05, log_10_ LDA score >3.5) were enriched or depleted across the pollution gradient. More specifically, members of Proteobacteria (affiliated to *Burkholderiaceae*, *Micromonosporaceae*, *Xanthobacteraceae*, and *Rhodocyclaceae*) and Bacteroidetes (affiliated to *Flavobacteriaceae*) were enriched at the low level of pollution. ASVs assigned to the families such as *Xanthobacteraceae*, *Dongiaceae*, *Xanthomonadaceae*, *Rhizobiaceae* and *Caulobacteraceae*, *Devosiaceae*, *Fibrobacteraceae*, *Pseudomonadaceae* were discriminative for roots of plants grown in the absence of pollution. In the roots of plants exposed to high pollution, the genera *Herminiimonas*, *Methylophilus*, *Cupriavidus*, *Novosphingobium*, and *Oligotropha* were enriched. According to Venn diagrams, a high number of ASVs is shared among the root communities exposed to different levels of pollution (**Figure 7**). Similarly, the high percentage of ASVs can be found in both plant compartments along the pollution gradient.

### qPCR

**FIGURE 8:**
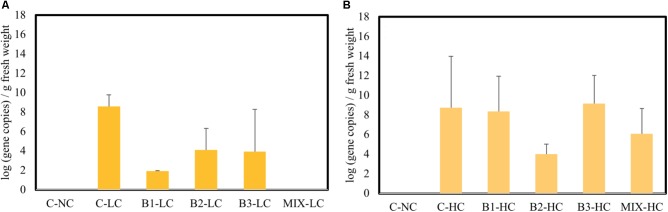
The abundance of *sul*I gene in the root interior of plants exposed to low **(A)** high **(B)** concentration mixture of pollutants (*n* = 3); data with asterisk are significantly different (*p* < 0,05) compared to the non-inoculated plants.

One sulfonamide gene (*sul*I) was detected in the endosphere of roots of plants exposed to mixed pollution, while it was not found in the roots of non-exposed plants (**Figure 8**). Fluoroquinolone resistant genes [*qnrA, qnrS*, and *aac(6′)-Ib-cr*] were not found in roots. At low concentrations of pollutants, the abundance of *sul*I gene showed significantly higher values in non-inoculated roots while it was not detected in plants inoculated with the consortium. When elevated concentration of pollutants was added to the aqueous phase, the abundance of SMX resistance genes increased in B1 and B3 inoculated plants as well as plants inoculated with the consortium after 21 days of incubation. In non-inoculated and B2 inoculated plants, the *sul*I abundance remained stable at the two levels of pollution.

## Discussion

Applying phytotechnologies to sites with mixed pollution is a complex issue, since possible interactions between organic xenobiotics and metals may occur as well as with the associated microorganisms. Moreover, the presence of different pollutants causes toxicity thus affecting plant growth and performance (Chirakkara et al., 2016). Taking into consideration that the presence of a mixture of pollutants is the case in almost every CW, new approaches to improve the wastewater remediation efficiency of CWs need to be investigated. For example, choosing the appropriate plant is a crucial issue; *J. acutus* has been characterized as a promising wetland plant for both, metals and organics remediation (Syranidou et al., 2017a).

Bioaugmentation with indigenous endophytic bacteria influenced positively metal concentration in plant tissues at both levels of mixed pollution; they significantly enhanced Zn concentrations inside the plant while they also increased Ni concentrations to a lesser extent (**Supplementary Figures S2–S4**). Earlier, it was also shown that bioaugmentation with endophytic bacteria stimulates the host to uptake metals (Ma et al., 2015, 2016b; Visioli et al., 2015) while at the same time no effect of metal accumulation was demonstrated by other studies (Mesa et al., 2015). Metal tolerant bacteria expressing plant growth properties (ACC deaminase, siderophores, IAA) were inoculated to *J. acutus* as they may enhance its ability to accumulate Zn and Ni in the internal tissues by reducing the stress caused by metal toxicity. Endophytic bacteria can alter the phytoextraction capacity of their host plant through several mechanisms; they can influence the uptake, translocation, accumulation, transformation, and detoxification of metals (Ma et al., 2016a). Often, the inoculation of bacterial strains possessing plant growth promoting traits along with tolerance to the target compound has a positive phytoremediation outcome (Rajkumar et al., 2009; Afzal et al., 2014). Our results corroborate the before-mentioned observations. After considering the total amount of metals accumulated in the whole plant biomass, it was demonstrated that (in almost all cases) significantly higher amounts of Ni and Zn were accumulated inside inoculated plants in comparison to non-inoculated ones. It is worth mentioning that the consortium had the most pronounced effect on the concentrations of all three metals in *J. acutus*: the consortium inoculated plants contained approximately three times higher Ni, two times higher Zn and 1.5 times higher Cd concentrations in comparison to the non-inoculated ones. Utilizing a variety of metal tolerant strains ensured that many PGP characteristics will be expressed since the single inoculants are able to produce a limited number of traits.

The bacterial community is an important factor in determining the removal rates in CWs (Meng et al., 2014), and is strongly affected in terms of functionality and diversity by the presence of helophytes (Fernandes et al., 2015; Button et al., 2016). The type and the concentration of pollutants are also significant factors that shape the plant associated communities; negative trends in diversity of endophytic bacteria have been described in response to increased levels of pollutants (Peng et al., 2013; Su et al., 2015). High metal concentrations were reported to have negative or adverse effects on the microbial community (Khan et al., 2010; Mucha et al., 2013). In the more sensitive environment such as CW in comparison to soils, multi-metal pollution caused changes in the bacterial community (Zhang C. et al., 2016). A significant negative correlation between the alpha indices of the root endophytic community and the metal concentration was observed (**Figure 1**). Moreover, the root communities were separated in response to the level of pollution; communities from the non-polluted treatments were clearly different from the others, whereas a slight overlap was observed between communities of low and high-polluted treatments (**Figure 2**). Similarly, significant differences were detected in the cultivable endophytic communities isolated from *Halimione portulacoides* across a gradient of metal(loid) pollution (Fidalgo et al., 2016). A significant dissimilarity has also been revealed between the DDE-exposed and non-exposed root endospheres of *Cucurbita pepo* (Eevers et al., 2016).

For the leaf endosphere, a convergence among the leaf composition has been demonstrated and it may be attributed to the lower metal concentrations in this plant compartment (**Figure 4**). This suggests that this compartment is affected to a lesser extent by the mixed pollution. However, the cultivable leaf endophytic communities of *Spartina alterniflora* were reported to vary among plants harvested from oil polluted sites (Kandalepas et al., 2015). Most likely, the limited dispersal possibilities of seeds along with the oil presence and stochastic phenomena contribute to this dissimilarity. Host plant, sampling site and time have been identified to be significant factors that influence the leaf community at non-polluted areas (Ding et al., 2013).

Proteobacteria dominated the cultured endophytic community of several wetland plants growing in CWs (Calheiros et al., 2016). In our study, the family *Sphingomonadaceae* exhibits the higher relative abundance within the root endophytic communities from high and low pollution treatments (**Figure 3**). This is not surprising since this family comprises of well-known genera for their ability to degrade a variety of organic pollutants (Acosta-González et al., 2015; Waigi et al., 2017). This family together with the *Methylophilaceae*, *Burkholderiaceae*, and *Xanthobacteraceae* are the marker families in the root microbiome at high pollution treatments (**Figure 6**). Members of the family *Methylophilaceae* has been found to exhibit higher abundances in metal polluted soils (Kou et al., 2018). Increase in the abundance of the families *Burkholderiaceae* and *Xanthobacteraceae* has been demonstrated in the enrofloxacin and ceftiofur acclimated communities from rhizosphere sediments of CWs (Alexandrino et al., 2017). It is important to mention that the abundance of ASVs affiliated with the family *Pseudomonadaceae* decreased significantly in the roots exposed to low and high pollution although this family is frequently isolated from plants at polluted sites (Afzal et al., 2014).

The plant itself in combination with the environmental parameters such as the type and concentration of pollutants can selectively enrich specific genotypes of endophytic bacteria (Siciliano et al., 2001). For example, the concentration of genes encoding for PAH-ring hydroxylating dioxygenases was stimulated in endophytic communities isolated from plants growing in more polluted areas (Oliveira et al., 2014). Moreover, the abundance of these genes was significantly higher in a root endosphere of plants exposed to phenanthrene in comparison to the control community (Hong et al., 2015). In our study, an antibiotic resistance gene to sulfamethoxazole was identified in the root endosphere of plants that were exposed to pollution and in the B3-inoculated strain while the presence of this gene was below detection level in the roots of non-exposed plants (**Figure 8**). Further experiments will reveal to what extent these genes can enhance the *in planta* sulfamethoxazole degradation, since the antibiotic can be dispersed within the plant parts. However, it is difficult to attribute this *in planta* enhancement of functional traits either to plant or to pollutant selection. For sure, the host plant plays an important role, influencing the endosphere composition and thus developing species specific relations in a polluted environment (Phillips et al., 2008; Lumactud et al., 2016). In unpolluted environments, it has been demonstrated that the host genotype affects the endophytic community to a lesser extent than the soil type or the soil microbial community (Bulgarelli et al., 2012; Lundberg et al., 2012). The 16S rRNA gene pyrosequencing of bulk soil, rhizosphere, and root samples of eight *Arabidopsis* ecotypes grown in two soil types revealed that among all the 778 OTUs, only 12 OTUs showed host genotype–dependent quantitative enrichment of the root endophytes (Lundberg et al., 2012).

Although the pollution has been characterized as a significant factor that influences the plant microbiome, inoculation of indigenous endophytic bacterial strains did not alter the endophytic community structure, which is in accordance with other studies (Conn and Franco, 2004). Despite the fact that differences were detected in the metal concentrations in the plants, inoculation of indigenous bacterial strains was not strongly influencing the community. In accordance with this observation, inoculation with *Burkholderia phytofirmans* PsJN to maize cultivars did not change the shoot and rhizosphere communities (Touceda-González et al., 2015). However, a strong effect on the soil microbial community has been observed after inoculation with a soil strain, while inoculation with a consortium had minor effects (Festa et al., 2016).

Until now, only few studies have investigated the effects of host genotype–dependent variation on the root bacterial microbiota profiles and the responses of endophytic communities to pollution with high-resolution techniques. In case of CWs, the studies mainly focus on the soil and rhizosphere compartment (Fernandes et al., 2015; Yi et al., 2016). This study contributes to the understanding of host-pollutant interactions and the endosphere which in turn should aid us in improving the efficiency of CWs. However, more studies are required to shed light on the underlying mechanisms that drive the interactions between plants and their bacterial community in response to increased levels of stress.

## Conclusion

Engineering the plant endosphere towards enhancing the efficiency of the host is expected to expand CW applications. Bioaugmentation with indigenous endophytic bacterial strains was shown to improve the metal phytoextraction potential of the wetland plant *J. acutus*. The increased concentration of the mixture of pollutants seemed a crucial factor that decreased the diversity and shaped the root endosphere communities while the inoculation effort had minor impact. Moreover, the diversity of the root communities showed a significant correlation with the metal concentrations in this plant part as well as the community composition, while the extent of this correlation varied among the inoculated plants. In contrast, the leaf communities seemed to remain unchanged across the pollution gradient the plants were exposed to. Specific ASVs were enriched in the root compartment in response to high levels of mixed pollution. However, it has not yet been examined whether plants growing in multi-polluted soils/water alter the survival potential of specific resistant and/or beneficial microbes. Thus, it is crucial to explore the diversity, distribution, and activity of endophytic microbial communities associated with various plant species in phytoremediation studies and monitor the changes induced due to pollution. To the best of our knowledge, this is the first study that uses high throughput analysis in order to elucidate the responses of endophytic communities to different levels of mixed pollution, including metals and emerging organic contaminants. More studies are needed to reveal the underlying mechanisms that drive the synergistic relationships between plants and their endophytic bacteria in order to exploit this symbiosis towards more robust and resilient phytoremediation technologies.

## Author Contributions

ES performed most of the experimental work and wrote the first draft manuscript. ST helped with the amplicon libraries preparation, the analysis of the metagenomic data, and with the discussion of the statistics. ES, ST, and IP monitored the colonization efficiency of the strains. MA performed the qPCR analysis. ES, ST, NW, DV, JV, and NK contributed in the design of the experiments. ST, NW, DV, JV, and NK helped with the proofreading, and revision of the manuscript.

## Conflict of Interest Statement

The authors declare that the research was conducted in the absence of any commercial or financial relationships that could be construed as a potential conflict of interest.

## Abbreviations

ARGs: antibiotic resistance genes
BPA: bisphenol A
CIP: ciprofloxacin
EOCs: emerging organic contaminants
SMX: Sulfamethoxazole
