# Supplementary material for: Responses of the Endophytic Bacterial Communities of Juncus acutus to Pollution With Metals, Emerging Organic Pollutants and to Bioaugmentation With Indigenous Strains
Source: Front Plant Sci. 2018 Oct 18;9:1526. doi: 10.3389/fpls.2018.01526 (PMC6200866; doi:10.3389/fpls.2018.01526)
Supplement: Supplementary file 1 [file Table_1.DOCX]

**Supplementary material**

**Responses of the endophytic bacterial communities of *Juncus acutus* to pollution with metals, emerging organic pollutants and to bioaugmentation with indigenous strains**

**Evdokia Syranidou^1.2^, Sofie Thijs^2^, Marina Avramidou^1^, Nele Weyens^2^, Danae**

**Venieri^1^, Isabel Pintelon^3^, Jaco Vangronsveld^2^ and Nicolas Kalogerakis^1,4^**

^1^ School of Environmental Engineering, Technical University of Crete, Chania, Greece

^2^ Centre for Environmental Sciences, Hasselt University, Diepenbeek, Belgium

^3^ Laboratory of Cell Biology and Histology, University of Antwerp Campus Groenenborger, Antwerp, Belgium

^4^Departemnt of Chemical Engineering, American University of Sharjah, Sharjah, UAE


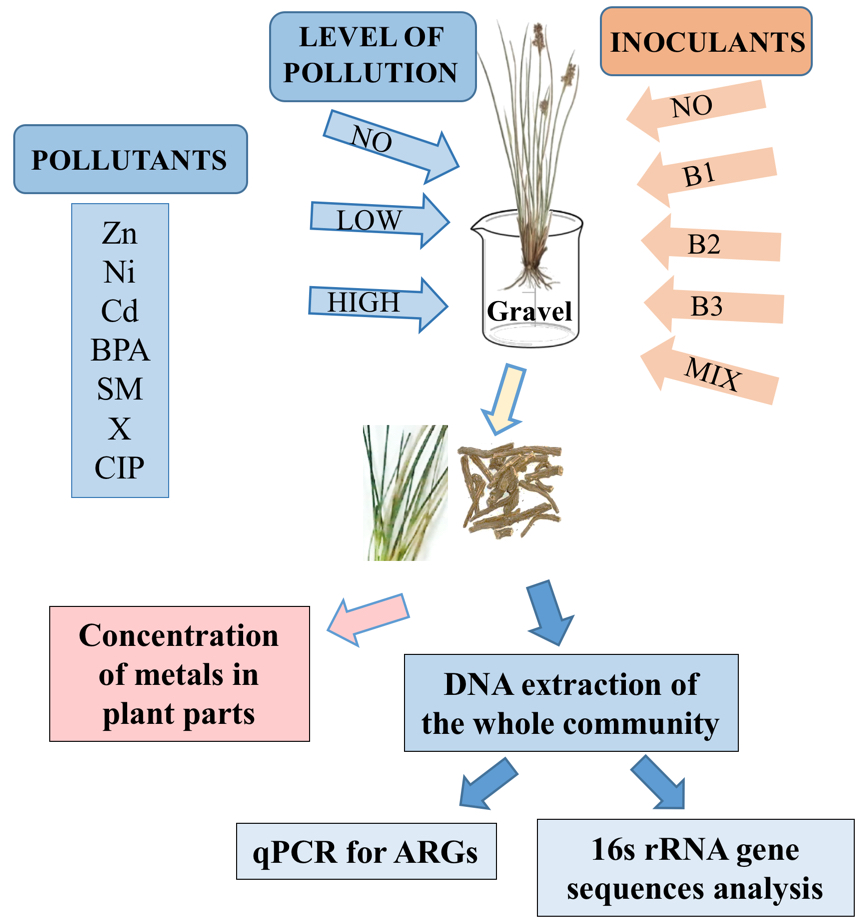


Figure S1. Schematic representation of the experimental design


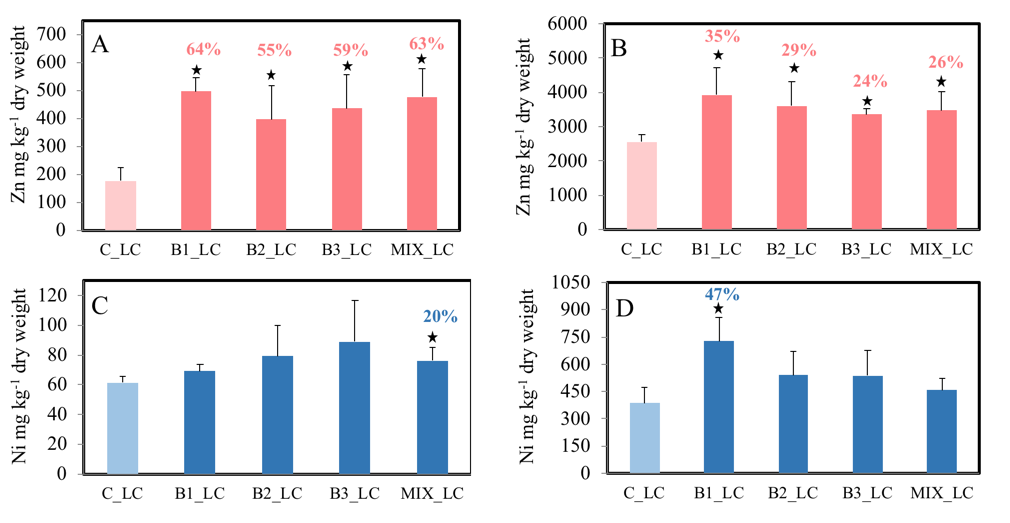


Figure S2. The Zn (A&B) and Ni (C&D) concentrations in the leaves (A&C) and roots (B&D) of *J. acutus* exposed to low concentrations of mixed pollutants (n=10); data with asterisk are significantly different (p<0,05) compared to the non-inoculated plants.


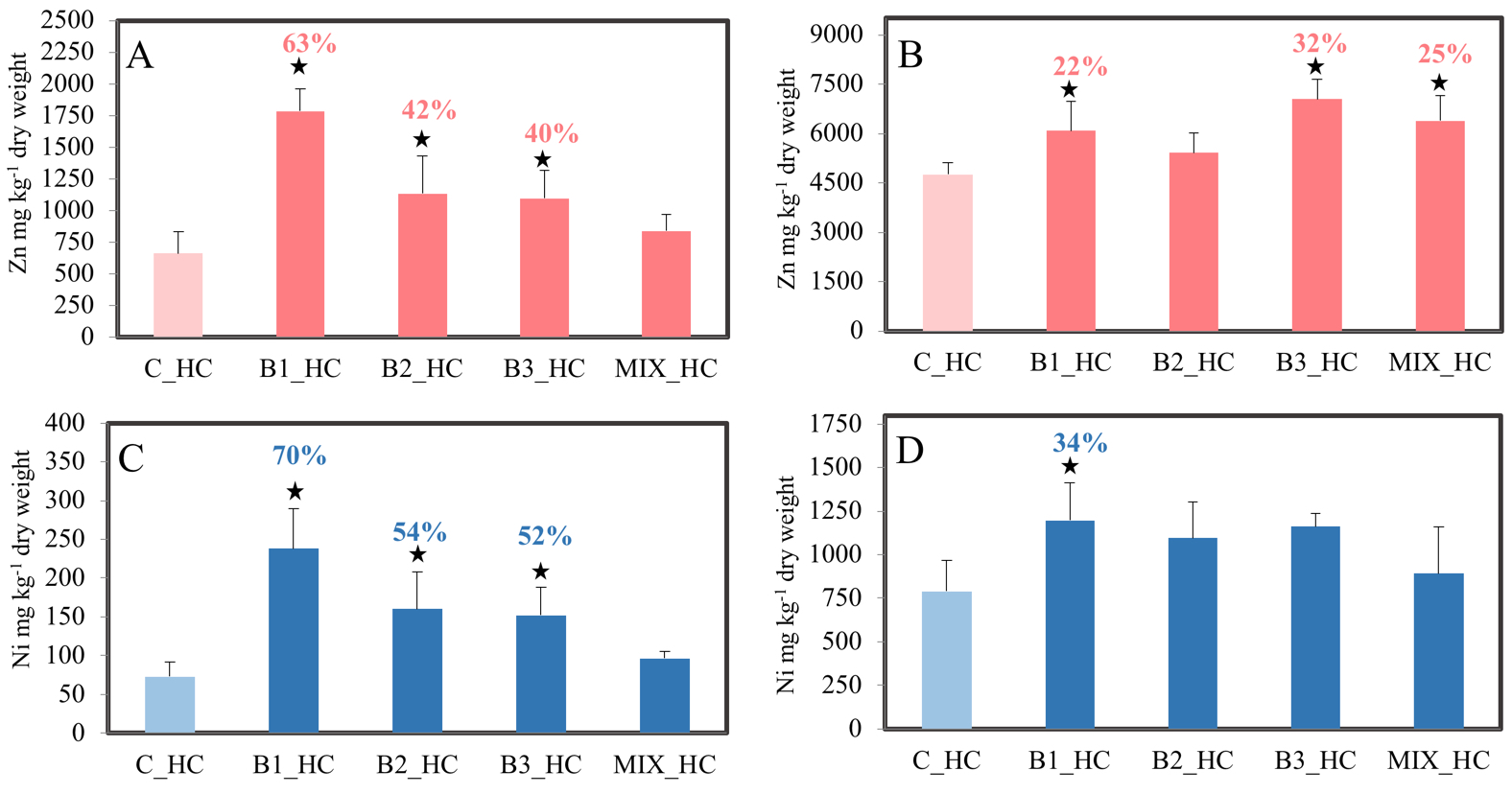


Figure S3. The Zn (A&B) and Ni (C&D) concentrations in the leaves (A&C) and roots (B&D) of *J. acutus* exposed to high concentrations of mixed pollutants (n=10); data with asterisk are significantly different (p<0,05) compared to the non-inoculated plants.


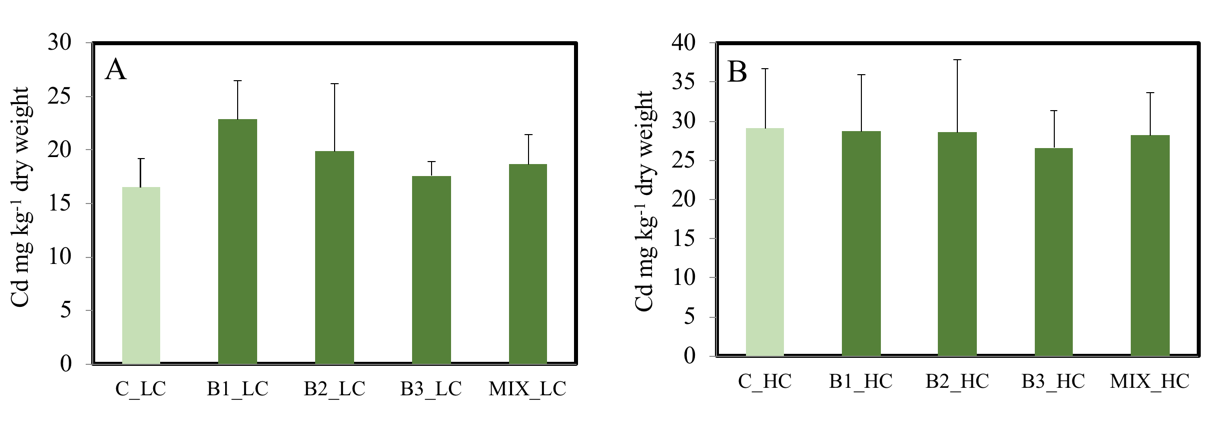


Figure S4. The Cd concentrations in the roots of *J. acutus* exposed to (A) low and (B) high concentration of pollutants (n=10); data with asterisk are significantly different (p<0,05) compared to the non-inoculated plants.


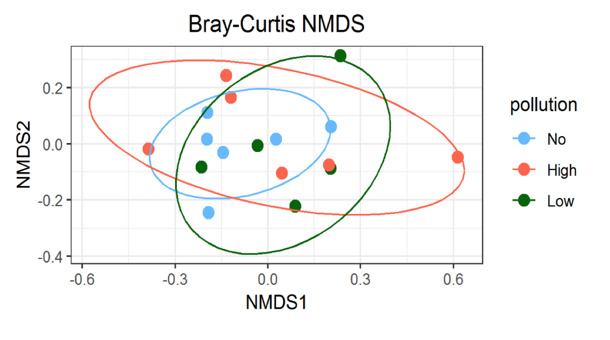


Figure S5. Similarity of the leaf endophytic communities across a mixed pollution gradient (A). Distance between the samples is based on similarity of the community composition visualized in non-metric multidimensional scaling (NMDS).
